# Supplementary material for: Large-area and efficient perovskite light-emitting diodes via low-temperature blade-coating
Source: Nat Commun. 2021 Jan 8;12:147. doi: 10.1038/s41467-020-20433-4 (PMC7794572; doi:10.1038/s41467-020-20433-4)
Supplement: Supplementary file 1 — Supplementary Information [file 41467_2020_20433_MOESM1_ESM.pdf]

Supplementary information

Large-area and efficient perovskite light-emitting diodes via low-temperature blade-coating

Shenglong Chu[1†], Wenjing Chen[1†], Zhibin Fang[1†], Xun Xiao[2], Yan Liu[1], Jia Chen[1], Jinsong Huang[2], and Zhengguo Xiao[1]*

[1]Hefei National Laboratory for Physical Science at the Microscale, Department of Physics, CAS Key Laboratory of Strongly-Coupled Quantum Matter Physics, University of Science and Technology of China, Hefei, Anhui 230026, China

[2]Department of Applied Physical Sciences, University of North Carolina at Chapel Hill, Chapel Hill, North Carolina, 27599, USA

[†]These authors contribute equally to this work

* Correspond to: zhengguo@ustc.edu.cn

Supplementary Figures

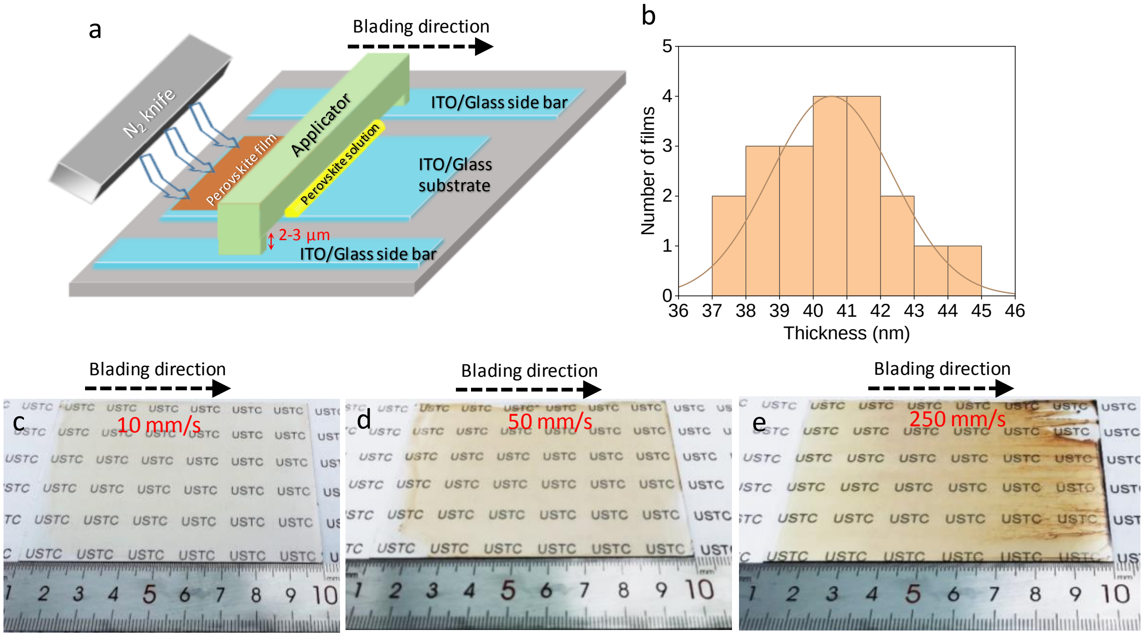

Supplementary Fig. 1 | Optimization of the blade-coating speed. a, Schematics of the blade-coating setup. **b**, Statistics of film thickness made from 0.04 M solution and coating speed of 150 mm/s. **c,d**, Photo images of blade-coated films with coating speed of 10 mm/s (**c**), 50 mm/s (**d**), and 250 mm/s (**e**). A N_2 knife was used for all films. The lower coating speed (e.g. 10 mm/s) can also results in uniform but very thin films, while higher coating speed of 250 mm/s results in non-uniform films due to the flowing of solution on the substrate. The substrate sizes are 6 cm × 9 cm.

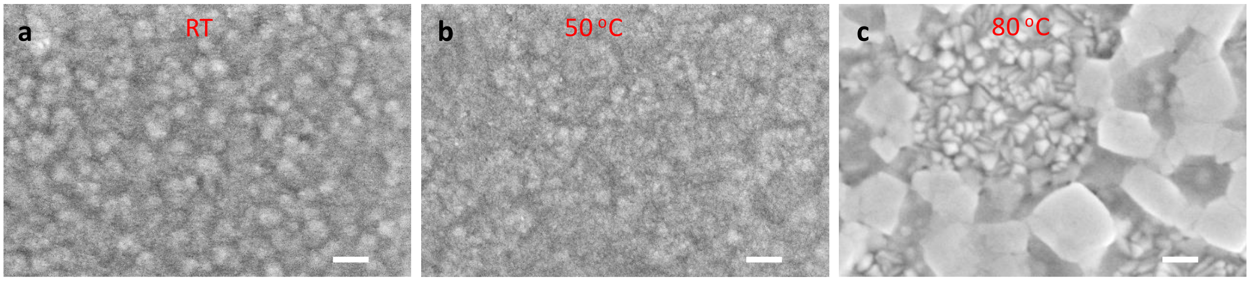

Supplementary Fig. 2 | Optimization of the blade-coating temperature. a-c, SEM images of perovskite films blade-coated at room temperature (**a**), 50 °C (**b**) and 80 °C (**c**). The coating speed for all films are 150 mm/s. The scale bars are 100 nm. The crystallization rate is slow at room

temperature even with the help of N_2 knife, resulting in rough films with larger grain sizes. On the contrary, if the coating temperature is too high (e.g. 80 ^oC), the film starts to dry before we apply the N_2 knife (Landau-Levich regime). And therefore, the film becomes very rough.

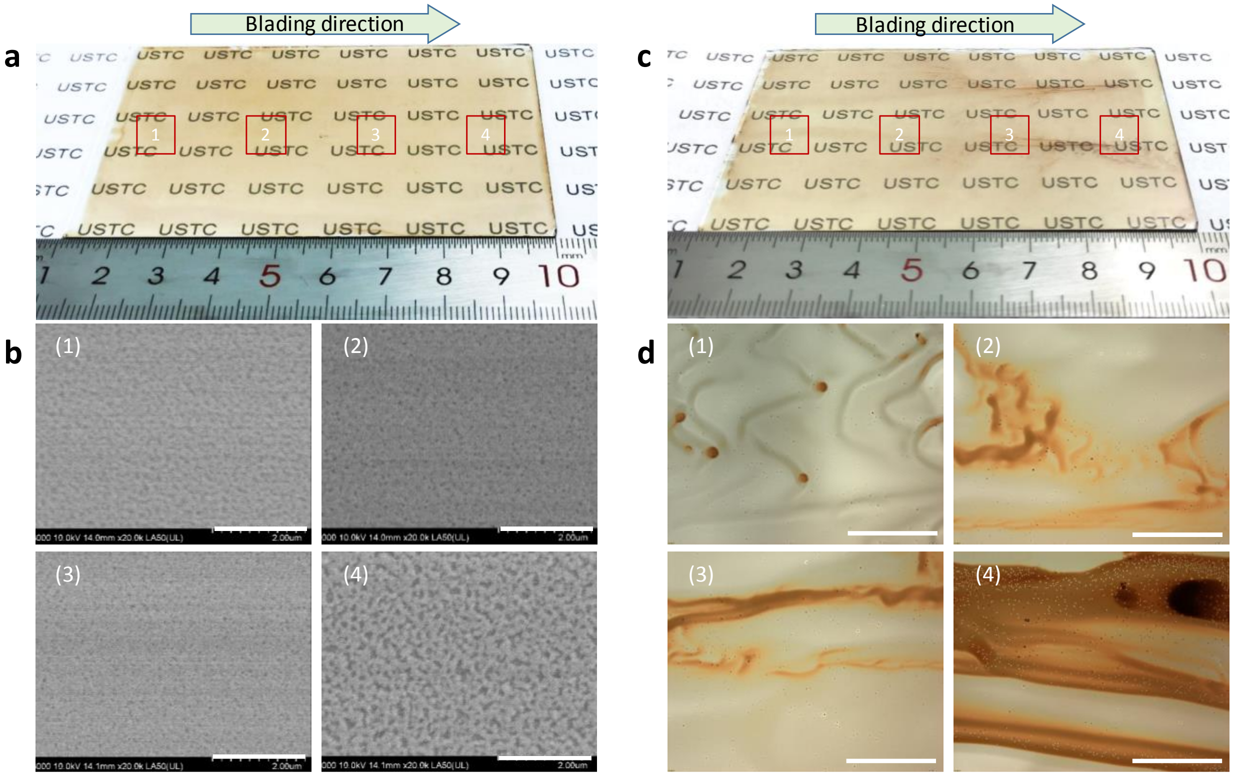

Supplementary Fig. 3 | Optimization of the N_2 knife pressure. a,b, A photo image of a blade-coated film fabricated using a N_2 knife pressure of 0.1 MPa (**a**), and SEM images taken at different positions (**b**). The scale bars are 2 μm. **c,d,** A photo image (**c**) and optical microscope images at different positions (**d**) of a perovskite film fabricated using a N_2 knife pressure of 0.4 MPa. The scale bars are 0.4 mm.

The film became very hazy if the N_2 knife pressure reduced from 0.2 MPa to 0.1 MPa, resulted from the slower evaporation rate of DMF solvent. SEM images show that the films are not uniform with many pinholes. When the pressure of N_2 knife was increased to 0.4 MPa, the N_2 knife drove perovskite wet film flowing on the substrate, resulting in a non-uniform film.

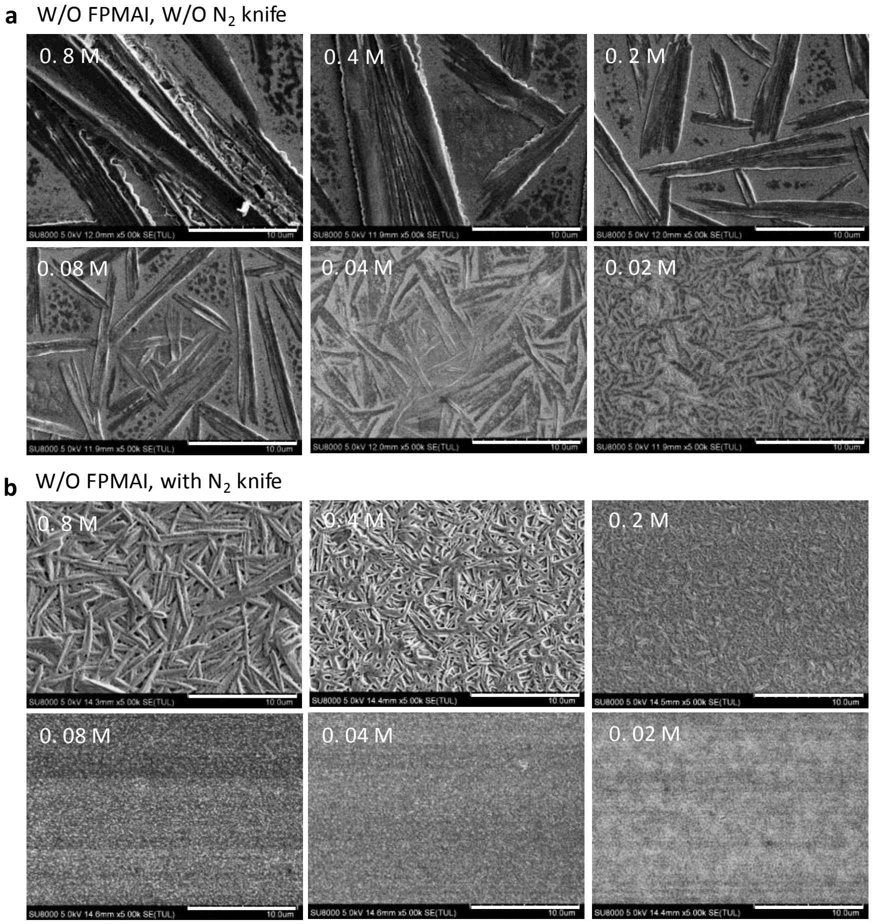

Supplementary Fig. 4 | SEM images of the doctor-bladed films fabricated W/O FPMAI or N_2 knife (**a**), and W/O FPMAI and with N_2 knife (**b**). The molar concentrations of the precursors are also marked in the images. The scale bars are 10 μm.

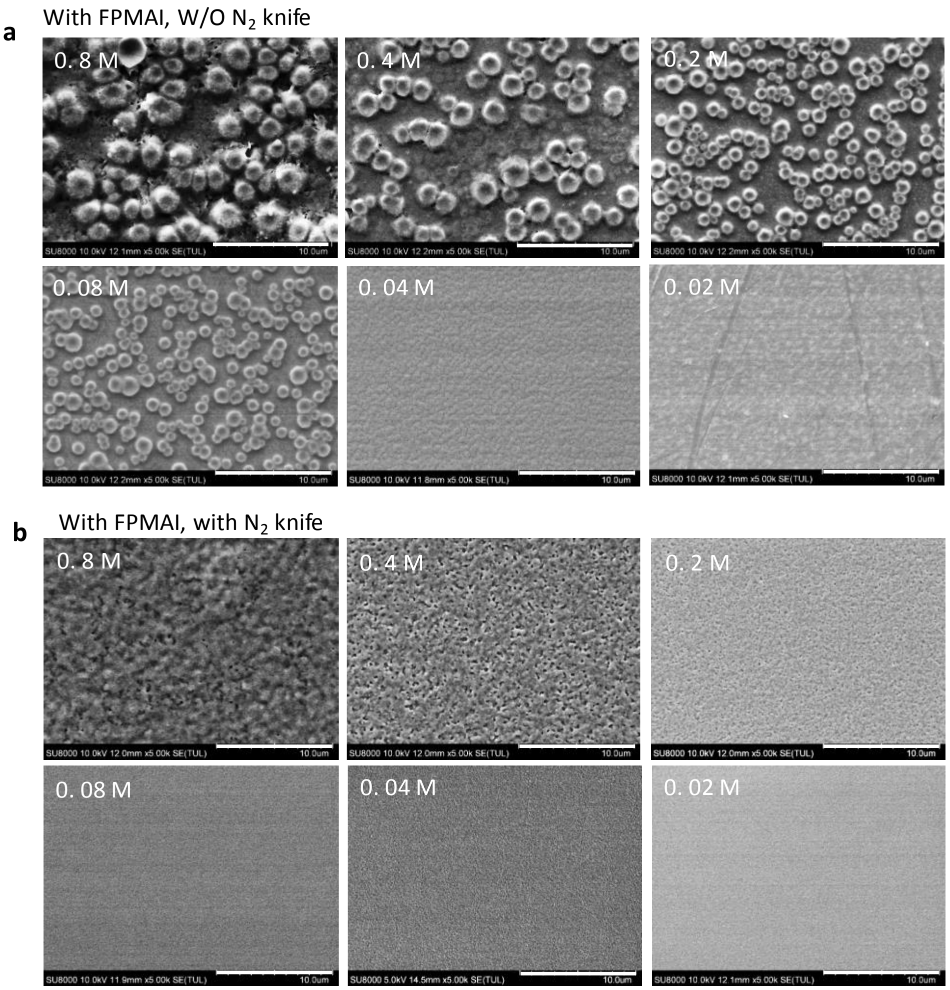

Supplementary Fig. 5 | SEM images of the doctor-bladed films fabricated with FPMAI and W/O N_2 knife (**a**), and with FPMAI and N_2 knife (**b**). The molar concentrations of the precursors are also marked in the images. The scale bars are 10 μm.

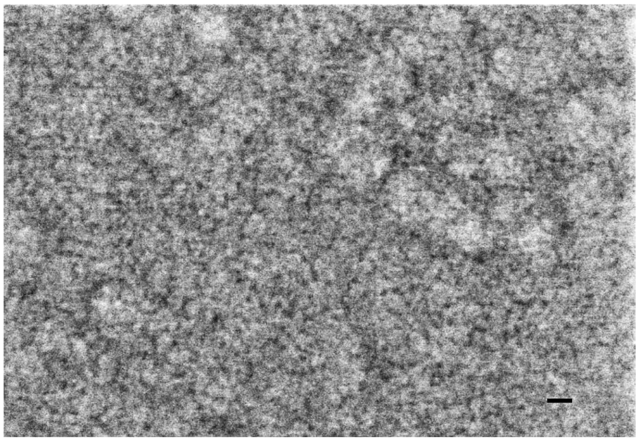

Supplementary Fig. 6 | A SEM top morphology image of the optimized perovskite film. The scale bar is 20 nm.

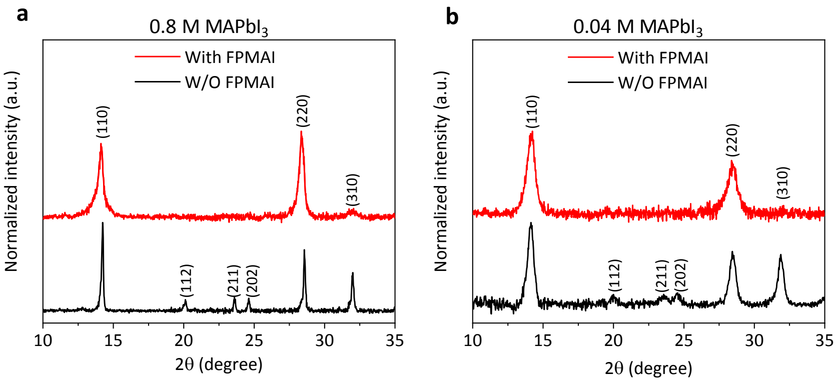

Supplementary Fig. 7 | XRD characterizations of both (**a**) thick (0.8 M, 770 nm) and (**b**) thin (0.04 M, 40 nm) films prepared with N_2 knife.

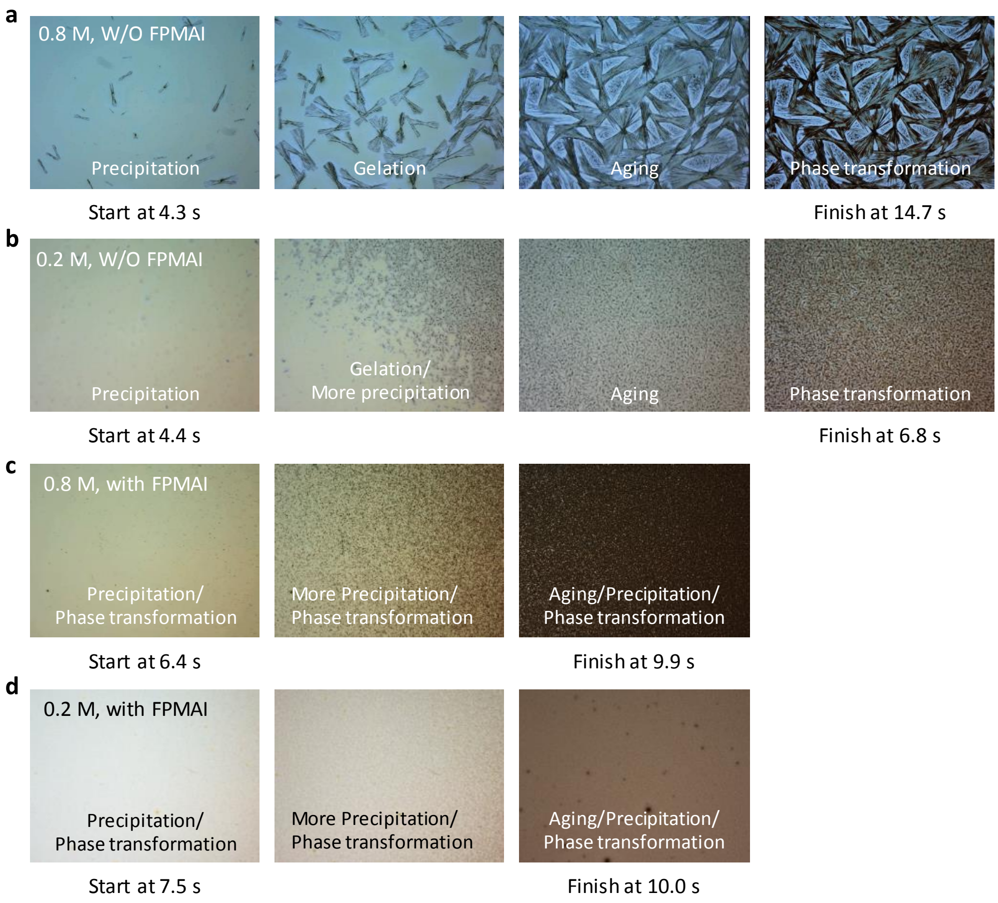

Supplementary Fig. 8 | Snapshots of the videos of the film forming process using 0.8 M precursor W/O FPMAI (**a**), 0.2 M precursor W/O FPMAI (**b**), 0.8 M precursor with FPMAI (**c**), and 0.2 M precursor with FPMAI (**d**). The picture sizes are 0.6 × 0.45 mm.

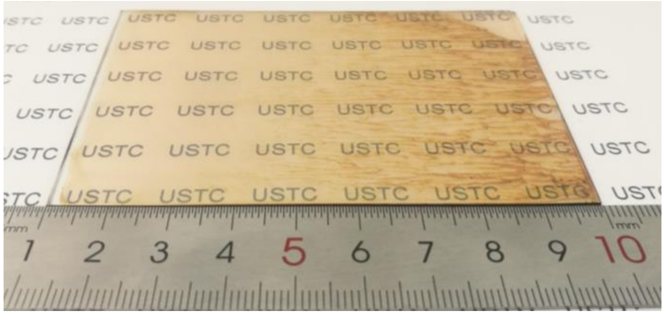

Supplementary Fig. 9 | A photo image of a non-uniform film fabricated using a 10 μm applicator.

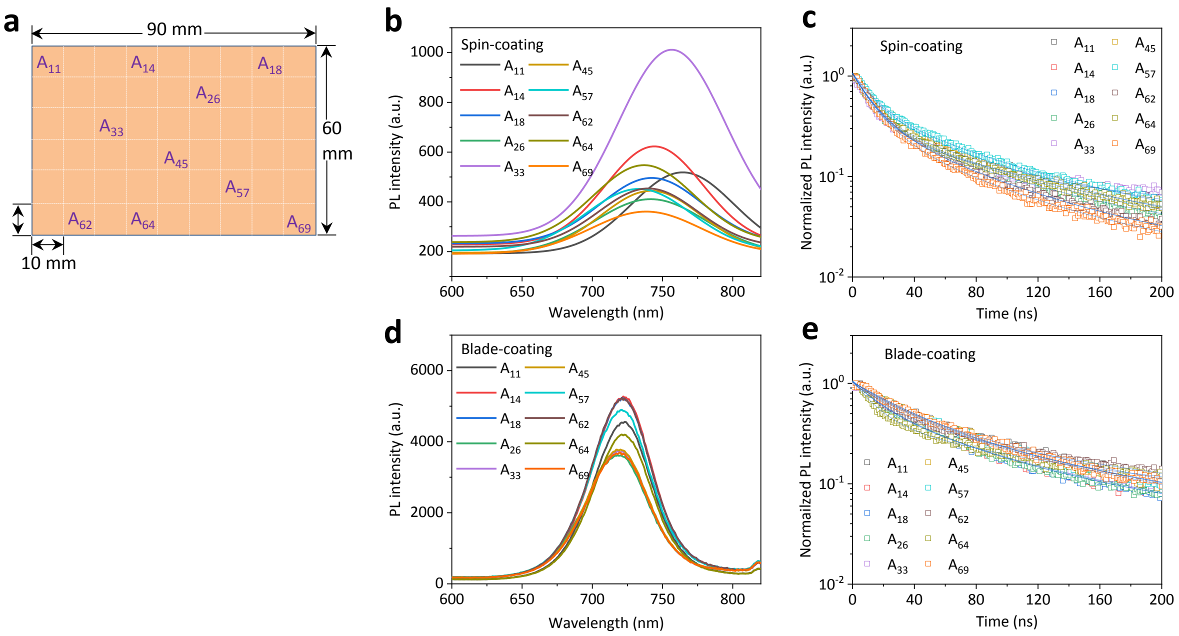

Supplementary Fig. 10 | Steady-state PL and TRPL characterizations of large-area films. a, A schematic of the large-area film divided into 1×1 cm^2 pieces. Each piece is marked as A_{xy} (x, y=1, 2, 3 …). **b-e**, Steady-state PL (**b,d**) and TRPL (**c,e**) of a large-area film fabricated by spin-coating (**b,c**) and blade-coating (**d,e**). The numbers of small-area films are marked in the figures.

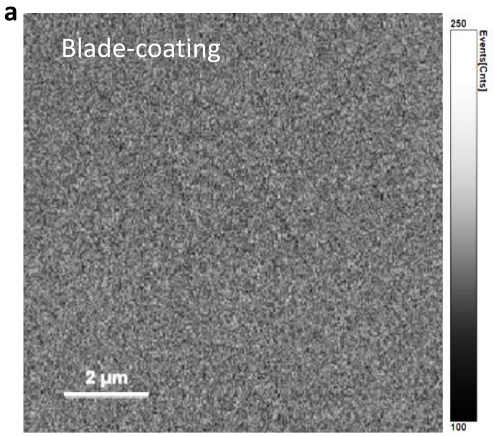

Supplementary Fig. 11 | Microscale optoelectronic characterization of perovskite films. a,b, Microscale PL intensity mapping of the blade-coated film (**a**) and spin-coated film (**b**).

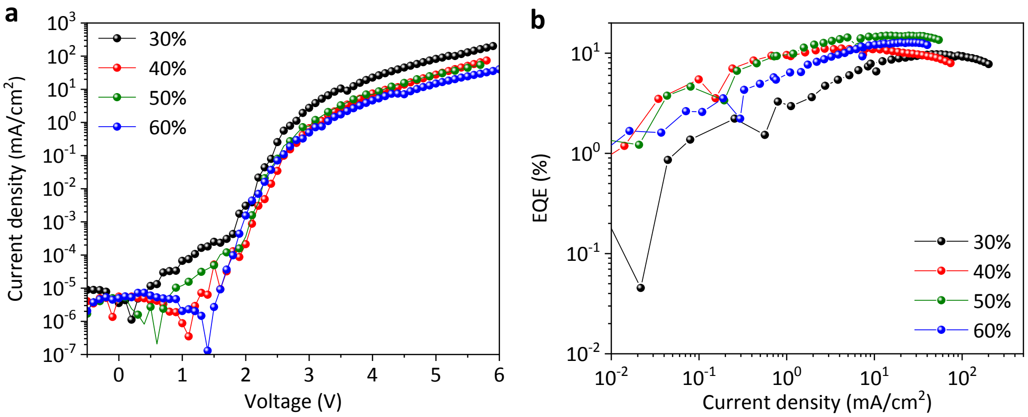

Supplementary Fig. 12 | Optimization of the FPMAI molar ratios. a,b, *J*-*V* (**a**) and EQE (**b**) curves of the PeLEDs with different molar ratios of FPMAI.

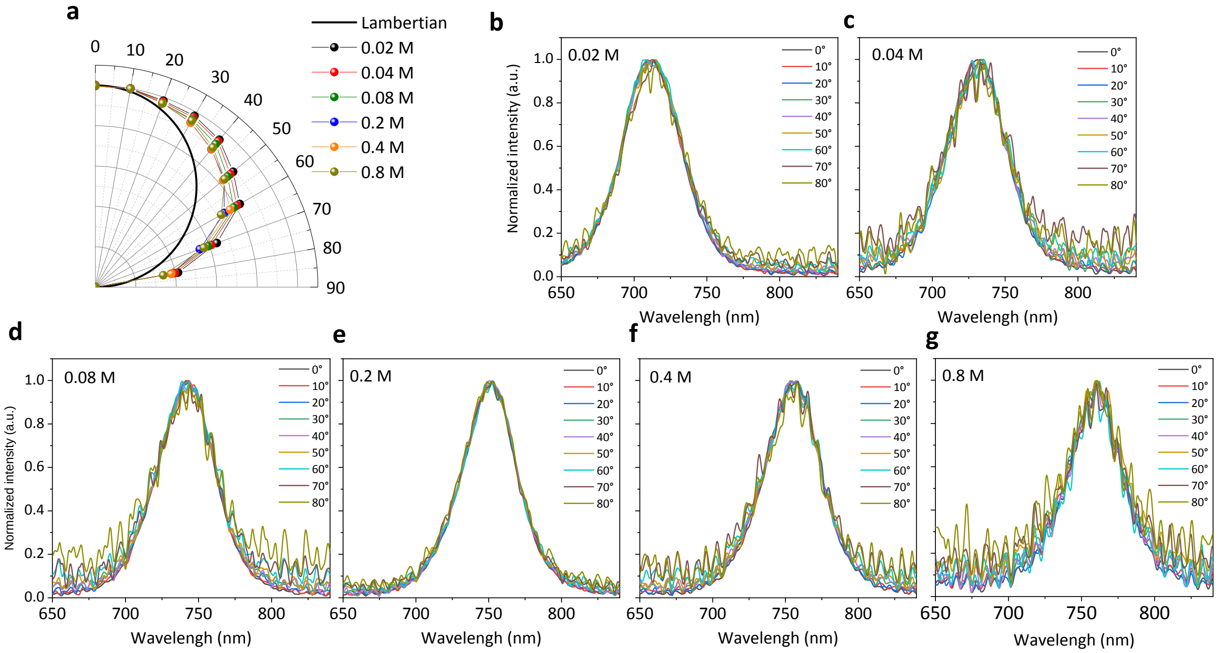

Supplementary Fig. 13 | Angular spectra and intensity profiles of PeLEDs. a-g, The angular intensity profiles (**a**), and spectra (**b-g**) of PeLEDs with different precursor concentrations. The PeLEDs are fabricated with 50% molar excess FPMAI and N_2 knife.

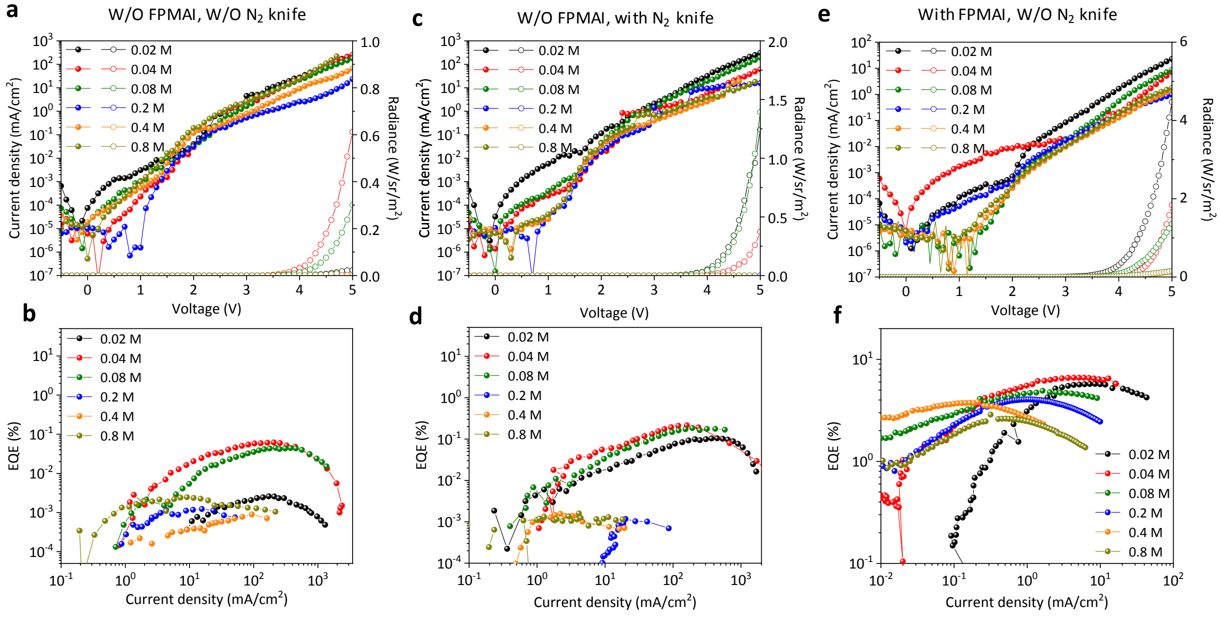

Supplementary Fig. 14 | Device performances of PeLEDs fabricated by different methods. *J-V-R* curves (**a,c,e**) and EQE curves (**b,d,f**) of PeLEDs fabricated W/O FPMAI or N_2 knife (**a,b**), W/O FPMAI and with N_2 knife (**c,d**), and with FPMAI and W/O N_2 knife (**e,f**).

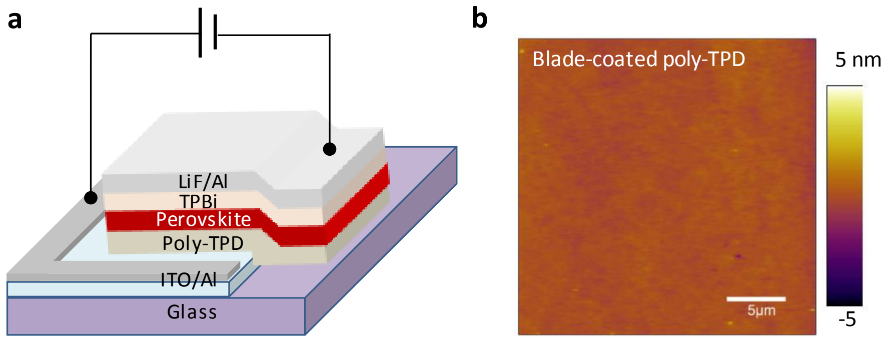

Supplementary Fig. 15 | Schematics of the PeLED structure and morphology of poly-TPD films. a, Device layout of large-area PeLEDs. **b**, AFM image of a blade-coated poly-TPD layer. The poly-TPD thickness is around 30 nm.

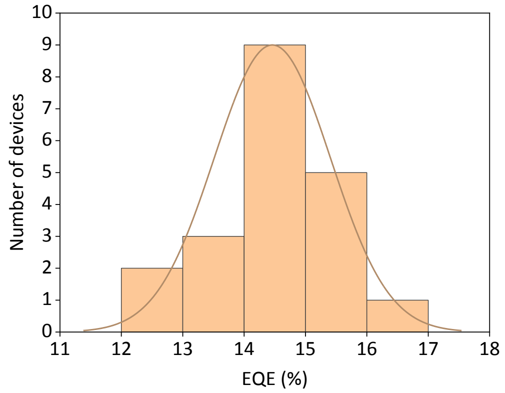

Supplementary Fig. 16 | EQE statistics of the PeLEDs made from 0.04 M solution.

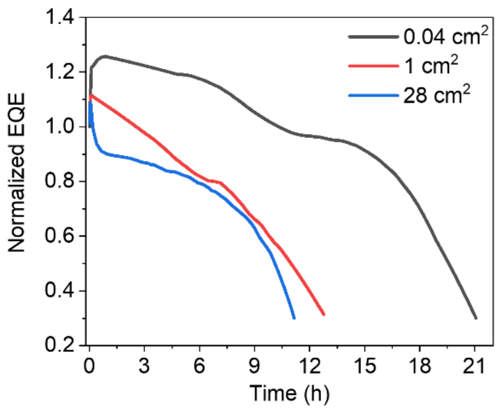

Supplementary Fig. 17 | Operation stability of blade-coated PeLEDs made from 0.04 M solution with different device areas. All the devices were measured at a constant current density of 3 mA/cm^2.

Supplementary Table 1. PLQY, $T_{average}$, K_{rad}, and K_{nonrad} values of large-area blade-coated films.

Blade-coating PLQY (%)

14.50	14.32	14.61	14.74	15.68	15.21	14.82	14.41	14.06
14.99	14.25	14.72	14.26	15.76	15.09	14.16	14.34	15.02
14.68	15.22	15.48	14.69	15.38	16.00	14.40	14.24	14.98
14.44	14.47	15.05	15.06	15.02	16.18	15.19	14.99	14.10
15.85	14.92	14.09	14.72	15.67	15.54	16.18	14.70	14.22
15.26	14.94	14.16	14.79	15.62	15.05	14.77	14.17	14.32

Y (cm) 1–6; X (cm) 1–9

Blade-coating $T_{average}$ (ns)

15.89	14.76	16.53	15.11	13.13	14.50	16.33	16.05	16.11
16.51	14.80	15.72	15.00	15.14	14.42	16.82	14.23	15.49
13.79	12.81	13.93	15.93	12.54	14.27	16.76	16.78	13.13
13.57	15.72	13.57	16.64	15.30	15.11	14.60	14.00	12.85
12.26	16.17	12.71	15.01	15.57	16.15	14.72	16.02	12.73
12.44	13.11	15.72	15.53	14.31	16.77	14.81	12.66	16.15

Y (cm) 1–6; X (cm) 1–9

Blade-coating K_{rad} (10^7 s^{-1})

0.91	0.97	0.88	0.98	1.19	1.05	0.91	0.90	0.87
0.91	0.96	0.94	0.95	1.04	1.05	0.84	1.01	0.97
1.06	1.19	1.11	0.92	1.23	1.12	0.86	0.85	1.14
1.06	0.92	1.11	0.91	0.98	1.07	1.04	1.07	1.10
1.29	0.92	1.11	0.98	1.01	0.96	1.10	0.92	1.12
1.23	1.14	0.90	0.95	1.09	0.90	1.00	1.12	0.89

Y (cm) 1–6; X (cm) 1–9

Blade-coating K_{nonrad} (10^7 s^{-1})

5.38	5.80	5.17	5.64	6.42	5.85	5.22	5.33	5.34
5.15	5.80	5.43	5.71	5.56	5.89	5.10	6.02	5.48
6.19	6.62	6.07	5.36	6.75	5.89	5.11	5.11	6.48
6.31	5.44	6.26	5.10	5.55	5.55	5.81	6.07	6.69
6.86	5.26	6.76	5.68	5.42	5.23	5.69	5.33	6.74
6.81	6.49	5.46	5.49	5.90	5.07	5.75	6.78	5.30

Y (cm) 1–6; X (cm) 1–9

Supplementary Table 2. PLQY, $T_{average}$, K_{rad}, and K_{nonrad} values of large-area spin-coated films.

Spin-coating PLQY (%)

2.45	3.30	4.69	4.48	3.59	3.52	4.57	3.59	3.88
3.10	4.94	6.07	6.88	6.12	4.97	5.93	4.20	3.78
4.72	5.40	6.11	6.56	6.74	6.79	5.05	4.62	3.48
4.44	5.60	5.21	6.34	6.00	5.64	5.54	5.36	3.44
5.00	5.85	5.00	5.30	5.64	4.99	6.81	5.81	3.70
3.37	4.15	3.35	4.95	5.15	4.85	3.72	4.31	3.01

Y (cm) 1–6; X (cm) 1–9

Spin-coating $T_{average}$ (ns)

9.14	7.10	6.92	7.59	4.46	4.79	6.97	6.98	6.55
8.94	6.51	9.39	7.83	4.87	4.57	7.15	4.87	7.50
7.93	5.85	7.09	8.60	8.98	4.45	7.12	4.53	5.57
4.54	4.77	8.81	7.30	9.60	5.13	4.87	7.96	5.86
8.72	7.83	7.67	8.66	8.80	5.06	6.84	5.13	4.58
9.31	6.88	5.33	9.37	8.81	5.85	7.94	4.55	4.77

Y (cm) 1–6; X (cm) 1–9

Spin-coating K_{rad} (10^7 s^{-1})

0.27	0.46	0.68	0.59	0.80	0.73	0.66	0.51	0.59
0.35	0.76	0.65	0.88	1.26	1.09	0.83	0.86	0.50
0.60	0.92	0.86	0.76	0.75	1.53	0.71	1.02	0.62
0.98	1.17	0.59	0.87	0.62	1.10	1.14	0.67	0.59
0.57	0.75	0.65	0.61	0.64	0.99	0.99	1.13	0.81
0.36	0.60	0.63	0.53	0.58	0.83	0.47	0.95	0.63

Y (cm) 1–6; X (cm) 1–9

Spin-coating K_{nonrad} (10^7 s^{-1})

10.67	13.62	13.78	12.58	21.60	20.13	13.68	13.82	14.67
10.84	14.61	10.01	11.89	19.27	20.78	13.16	19.69	12.84
12.02	16.17	13.24	10.86	10.38	20.95	13.33	21.05	17.32
21.04	19.79	10.76	12.84	9.79	18.41	19.39	11.88	16.48
10.89	12.02	12.39	10.93	10.72	18.79	13.62	18.36	21.04
10.38	13.93	18.14	10.15	10.76	16.26	12.12	21.01	20.32

Y (cm) 1–6; X (cm) 1–9
