# Supplementary material for: Large-area and efficient perovskite light-emitting diodes via low-temperature blade-coating
Source: Nat Commun. 2021 Jan 8;12:147. doi: 10.1038/s41467-020-20433-4 (PMC7794572; doi:10.1038/s41467-020-20433-4)
Supplement: Supplementary file 3 — Description of Additional Supplementary Files [file 41467_2020_20433_MOESM3_ESM.pdf]

Description of Additional Supplementary Files

File Name: Supplementary Movie 1
Description: Video of the film formation process for 0.8 M without FPMAI.

File Name: Supplementary Movie 2
Description: Video of the film formation process for 0.2 M without FPMAI.

File Name: Supplementary Movie 3
Description: Video of the film formation process for 0.8 M with FPMAI.

File Name: Supplementary Movie 4
Description: Video of the film formation process for 0.2 M with FPMAI.
